# Determining optimum assembly zone for modular reconfigurable robots using multi-objective genetic algorithm

**DOI:** 10.1038/s41598-024-84637-0

**Published:** 2025-01-03

**Authors:** Ravikiran Pasumarthi, S. M. Bhagya P. Samarakoon, Mohan Rajesh Elara, Bing J. Sheu

**Affiliations:** 1 Engineering Product Development Pillar, Singapore University of Technology and Design, Singapore, 487372 Singapore; 2 Department of Electronics Engineering, College of Engineering, Chang Gung University, Taoyuan City, 330 Taiwan

**Keywords:** Electrical and electronic engineering, Mechanical engineering

## Abstract

Reconfigurable modular robots can be used in application domains such as exploration, logistics, and outer space. The robots should be able to assemble and work as a single entity to perform a task that requires high throughput. Selecting an optimum assembly position with minimum distance traveled by robots in an obstacle surrounding the environment is challenging. Therefore, this paper proposes a novel approach for optimizing the assembly zone of modular robots in heterogeneous obstacle environments. The method uses a multi-objective Genetic Algorithm (GA) to minimize total travel distance and individual distance disparities. Utilizing the A* algorithm for path planning ensures efficient navigation. A generic kinematic model enabling holonomic locomotion with any reconfiguration and a new modular robot design are also introduced. Hardware experiments have been conducted to validate the kinematic model’s applicability for holonomic navigation across different robot configurations. Simulations and physical experiments demonstrated the effectiveness of the proposed method in determining assembly zones, with GA outperforming multi-objective pattern search and random selection in terms of total distance and individual distances traveled by the robots.

## Introduction

Reconfigurable modular robots are used in wide-ranging applications across diverse domains, including exploration^1^, cleaning^2^, education^3^ and outer space^4^. Among these, modular reconfigurable robots are known for their unique capability to assemble and disassemble, enabling them to adapt to specific tasks. This modularity enables these robots to reconfigure their structure in response to changing operational requirements, making them highly versatile in various scenarios^5^. There are different existing modular robots such as snake structuring robots^6^, 3-dimensional structure formation robots^7^, and modular self reconfigure manipulator robots^5^. Modular robots are adaptive for multiple applications and perform tasks more efficiently than conventional single-unit robots. Since the robots are interchangeable, the units can be replaced with other units. This enhances the robustness of the robot in usage. With these features, the modular robot can be used in a wide range of applications.

The modular robots are equipped with an advanced assembly mechanism that can also exchange power with the modules that are being docked in order to enable opportunities like maneuvering tasks, multi-terrain control applications, and adaptive 3-D structural robot formation. To achieve reconfiguration, modular robots employ a variety of assembly and disassembly methods, including magnetic connections, mechanical locking, and electro-controlled locking. In magnetic connections, conventional ferromagnets or electromagnets are employed to dock and assemble the robot units, ensuring a swift and efficient assembly process. The magnetic locking mechanism expedites the assembly and disassembly processes and introduces the potential for wireless power-sharing functionalities^8^. Mechanical docking, on the other hand, relies on intelligent mechanical design arrangements to seamlessly dock and assemble the robot components. This method is cost-effective and does not necessitate complex electronic components^9^. Electro-controlled locking employs servo motors or other relevant actuators to enable precise locking with another unit during assembly. Solenoid units can achieve rapid locking, while servo motors provide the precision required for intricate assembly tasks^10^. This diverse range of assembly methods ensures flexibility and adaptability in modular robot configurations, catering to various application scenarios.

Modular robots consist of multiple interchangeable units, resulting in changes in both size and shape. Consequently, maintaining consistent control over the robot is challenging due to its varying configurations. In other words, a robot’s kinematic model varies with different configurations of the robots. For example, a robot with a 4-wheel module can combined with another sort of robot and become an 8-wheel robot. The kinematics of the robot will change. There are many systems and research in kinematic modeling for different driving mechanisms such as regular 2-wheeled robots with convectional wheels, omnidirectional wheels, and ball wheels^11^. The wheeled robot kinematics should be closed loop to get control of the motions, such as connecting the encoders and IMU for localization for the proper usage of kinematics and the model built with these necessary apparatus. The proper filtering with offsets in feedback and deriving the transformation matrix in avoiding the error is introduced in^12^. Conventional wheels are being used in the existing robot. However, for more maneuvering of the robot, the robot can be adapted with the mecanum wheels, which enable the robot to move in holonomic motion^13^.

Efficient path planning is also an essential requirement for a set of modular robots operated in an environment^14^. Typically, path planning algorithms are developed to plan the path from a robot position to a targeted location by avoiding obstacles. An algorithm with more efficiency by optimizing the distance and time objectives was studied in^15^. To achieve efficiency optimization of one object or multi-object is needed. The article ^16^, mentioned the path planning and the comparison results of the optimization techniques. There are many algorithms have been used for robot path planning. Optimization techniques such as Genetic Algorithm (GA)^17^, particle swarm optimization^18^, grey wolf optimizer^19^, Pattern Search (PS)^20^, and other path planning algorithms such neural networks^21^, reinforcement learning^22^ have been used for both static and non-static obstacles environments. Collision-free multi-robot path planning in controlled environments using the A* algorithm with the co-evolutionary algorithms has been introduced in^23^. The work enhanced the pathfinding by using collision-free A* routes, reducing the search space by restricting the mutation points and tuning the probabilities of the movements to be considered in the mutation operator. In^24^, a transformer structure into policy neural networks has been introduced to enhance the ability of policy neural networks to extract features that facilitate collaboration between robots. The work^25^ proposed a coevolution-based particle swarm optimization method for multi-robot path planning. The introduced particle swarm optimization improves a widely used standard particle swarm optimization algorithm with the evolutionary game theory. Although the above research work was conducted for path planning for multi-robots, efficient assembly zones for multi-robot or modular robots are yet to be explored.

Robots used in exploration, logistics, and firefighting are scattered in a considered environment. Most of the time, these robots require assembling each other to strengthen themselves to perform certain tasks. For example, logistics robots must assemble each other to carry a heavy load. Since the robots are scattered away in a given environment, they need to find an efficient way of navigating every robot to an assembly zone. Therefore, this assembly zone should be an optimum zone for improving the efficiency of the robots. In order to find an optimum assembly zone, the robots’ total distance traveled should be minimized, and equal distances should be maintained to reach the zone. Although many path-planning strategies have been developed for reconfigurable modular robots, the identification of optimum assembly zones has not been explored before. Therefore, this paper proposes an optimum assembly zone determining using GA for modular robots in obstacles surrounding environments with different numbers of robots. Furthermore, the paper introduces a generic kinematic model for assembly with different configurations. The rest of the paper follows; The section “Modular Robot Platform” introduces the modular robotic platform with its architecture. The section “Kinematic Modeling” discusses the kinematic model for different configurations, and the section ‘Optimum Location for Modular Robots to Assembly” introduces the optimum zone for modular robots to assemble using GA. The section “Experimental results” discusses the simulation results and the real experiment results, and the section ’Conclusion’ concludes the work.

## Modular robot platform

**Fig. 1:**
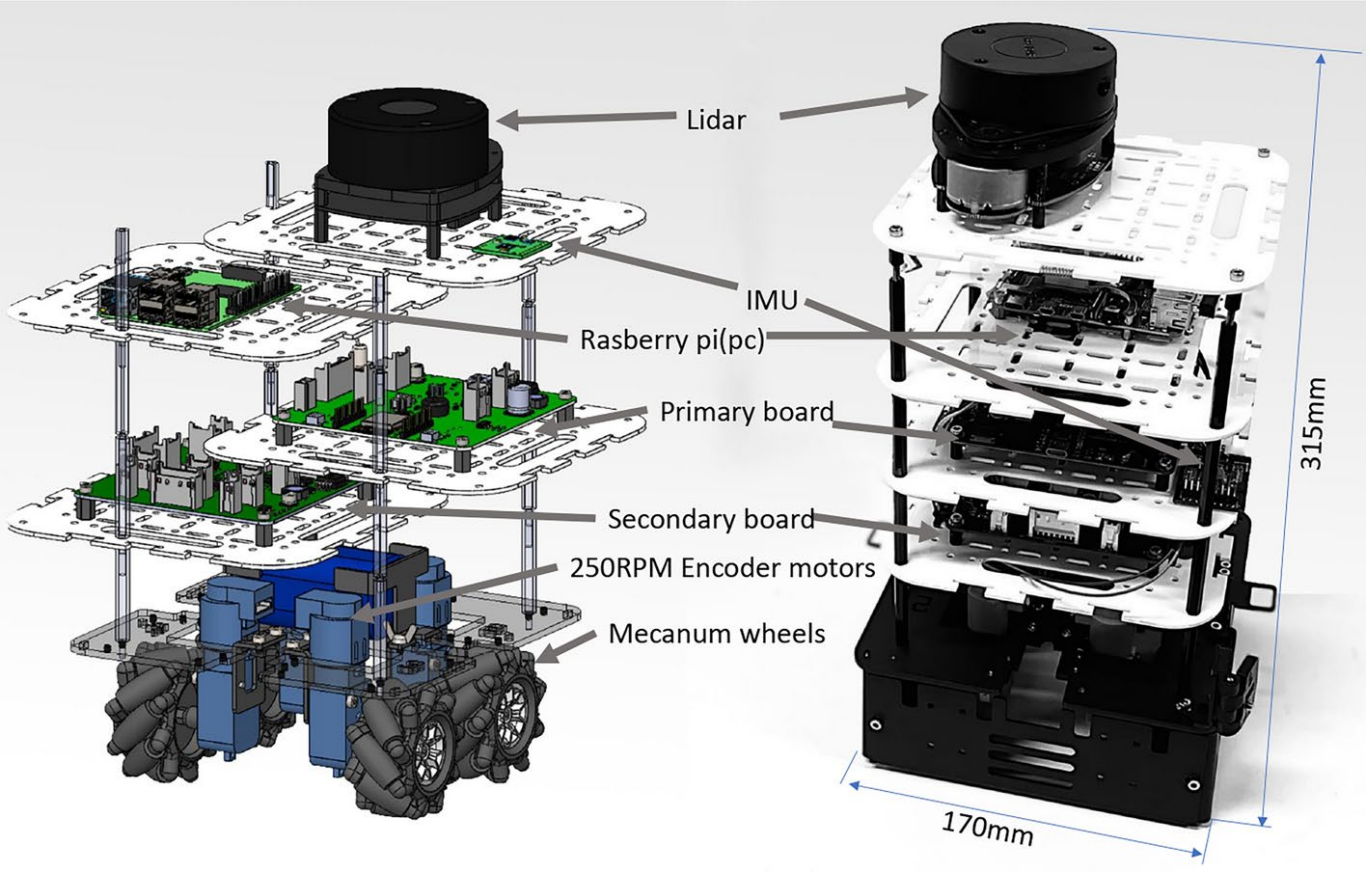
Smorphi-modular robot platform.

The Smorphi modular robot shown in Fig. 1 is designed with a footprint of 170 mm $\times$ 170 mm $\times$ 315 mm (width $\times$ length $\times$ height). The robot consists of mecanum wheels, which allow it to have holonomic motions. The motors used are 250RPM encoder motors, which have the advantage of generating odometry data. The robot has design features to enable the flexibility to accommodate additional sensor layers, stacked in the following order from bottom to top: base platform, secondary (driver board), primary board, PC (Raspberry PI), IMU, and Lidar. The robot’s primary board is designed to adapt multiple sensors, and it comes with the ESP32 chip, which has WiFi and Bluetooth communication enabled.

**Fig. 2:**
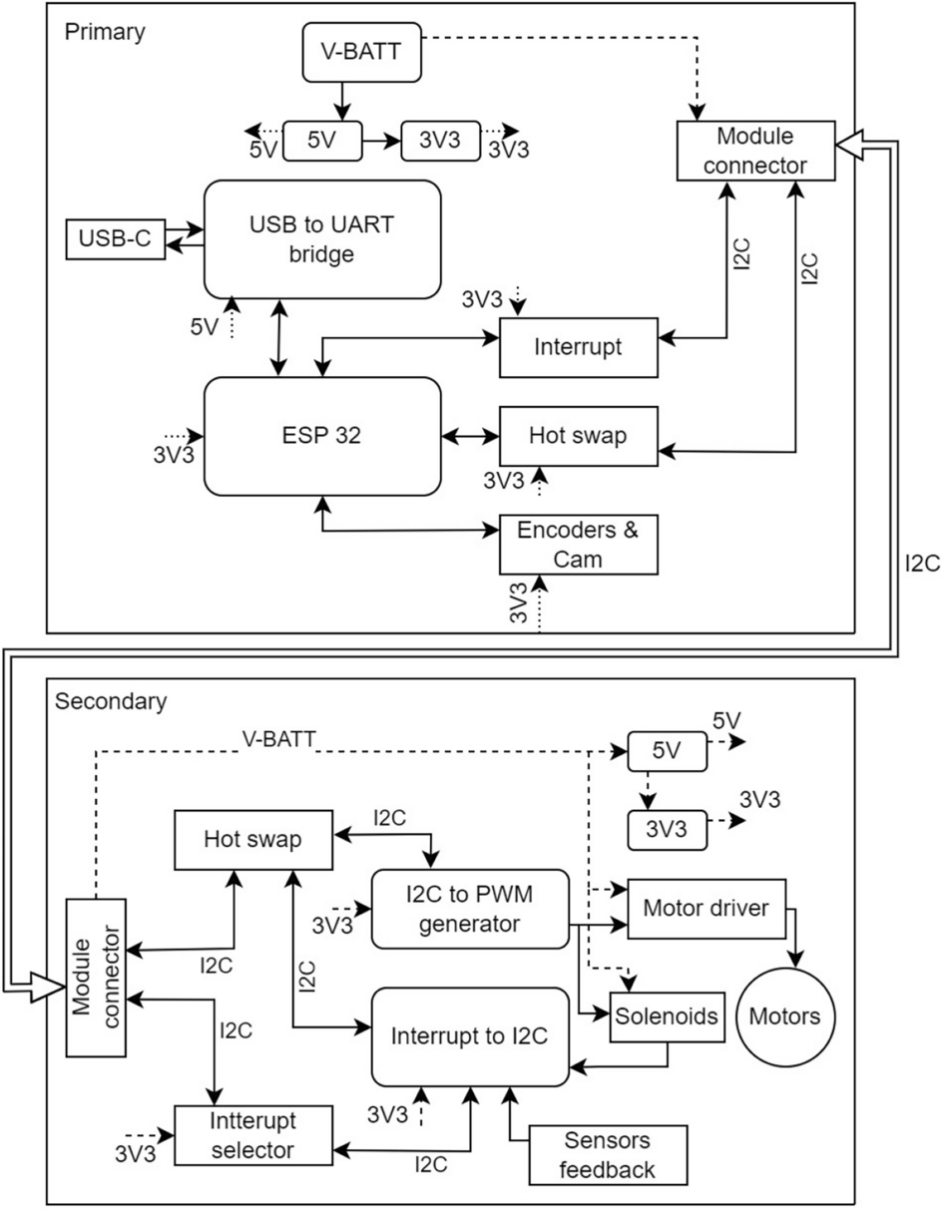
Electronic architecture.

### Electronic system architecture

The overall electronic architecture flow is illustrated in Fig. 2. The robot is powered with a 12V power supply. This 12 V power sully is successfully connected to two voltage regulators, 5 V and 3.3 V. The primary board of the robot is embedded with a microcontroller (ESP32) capable of generating instructions for the secondary board based on the provided code. The communication between the board follows the i2c protocol. The secondary board with motor driver and sensor interrupt generator decrepit instructions received and generated instructions to run the motors. Furthermore, it sends the encrypted feedback from the sensors, motors, and docking mechanism(an electro-mechanical mechanism). This integration of primary and secondary components interfaces with a PC (Raspberry Pi) via serial communication, enabling high-level control of the robot through instructions generated by the PC. The PC collects feedback from Lidar, IMU, and wheel encoders, leveraging this data to compute and execute autonomous tasks.

### Software architecture

**Fig. 3:**
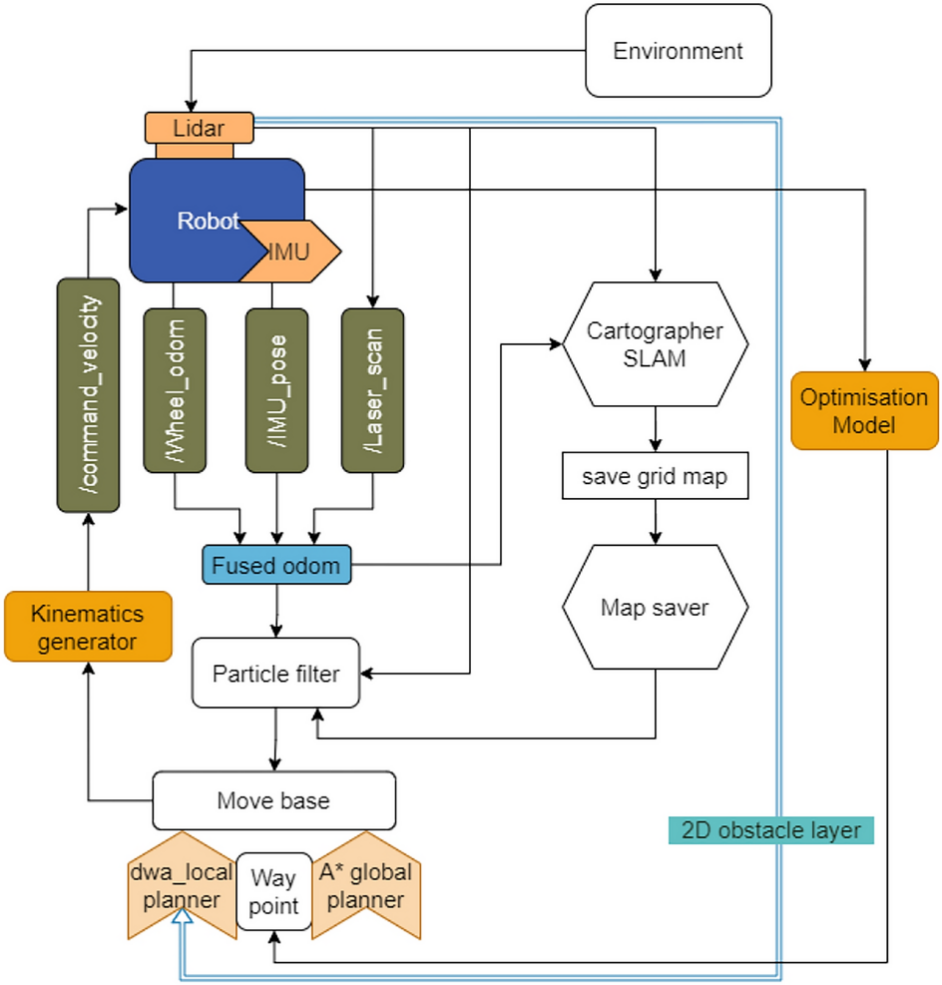
Software architecture.

The Smorphi robot can map and autonomous navigation using wheel odometry from the encoders embedded with the motors, 2D Lidar, and IMU. The overall software architecture of the robot is depicted in Fig. 3. The inputs from these Odom inputs were processed in the form of IMU_pose, Laser_scan, and wheel_odom, which will be fused for error correction through a particle filter. For mapping, the laser scan will be passed through the cartographer map, and the cartographer does the mapping and localization simultaneously, also known as the SLAM algorithm (Simultaneous mapping and localizing). On the other hand, the robot’s optimization model passes the assembly zone to the mobile base and generates the waypoints using the A* algorithm. The robotic system must adhere to a path defined by waypoints derived from an optimization algorithm, subsequently processed by the kinematics generator. This generator computes the necessary command_vel for the robot’s motors, ensuring precise velocity control during operation. With autonomous navigation, the robot can move from the robot position at a given time to the given goal point. This can be achieved by pre-mapping the environment using the 2D Lidar. The global and local planners generate the goal path for the robot to follow to reach the location, avoiding the obstacles on the map.

### Docking mechanism

**Fig. 4:**
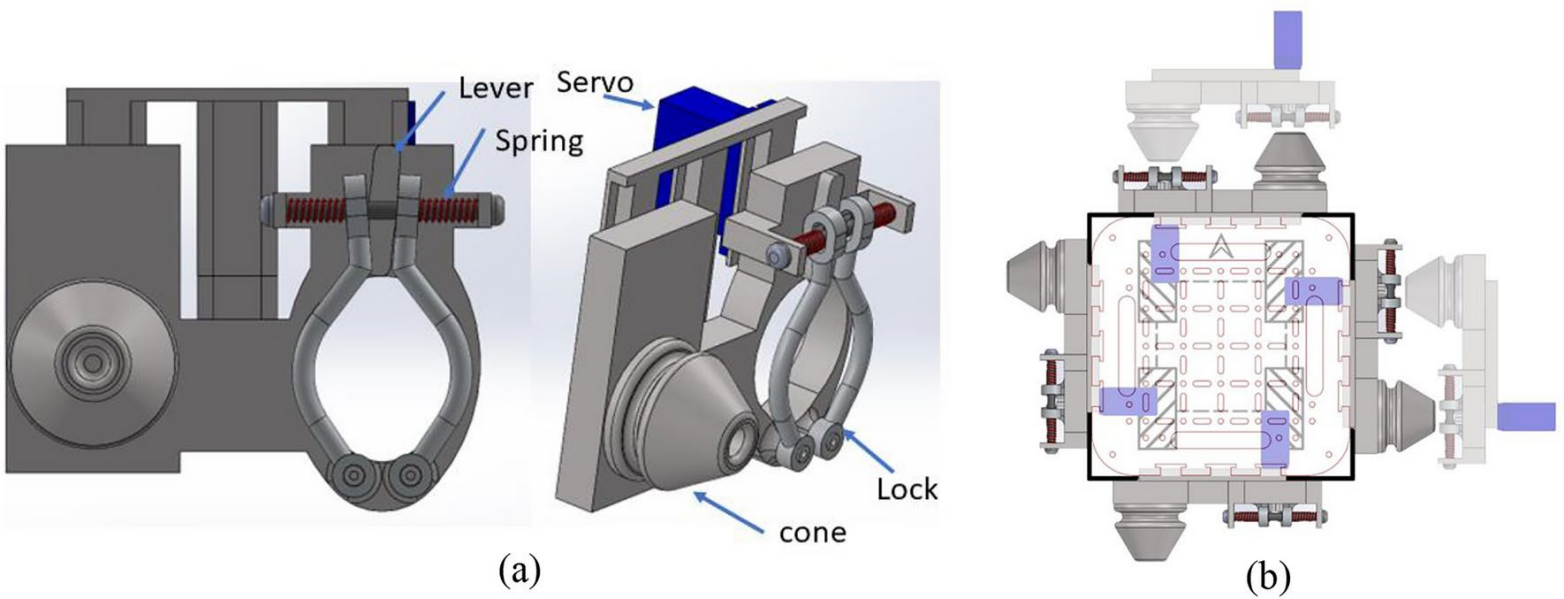
(**a**) Docking mechanism design, (**b**) Gender less feature of the docking mechanism designed.

To accomplish specific tasks, modular robots must undergo reconfiguration, morphing into various shapes by docking together. Therefore, we have developed an efficient and versatile docking system, as depicted in Fig. 4 for the Smorphi robot. The docking mechanism shown in Fig. 4a has been designed to facilitate effortless docking, even in scenarios where robots may be slightly misaligned due to variations in terrain. This is achieved by utilizing a cone and a mechanical locking system, complemented by incorporating a spring. The spring exerts a constant force, pushing the lever inward. When the cone makes contact, it prompts the lock to disengage, allowing it to be securely locked in place. To release the cone, a servo motor assists in the separation of the lever and enables the locked robots to move apart. This innovative combination of cone, mechanical locking, and spring-supported mechanisms ensures a reliable and secure docking process and addresses potential misalignments, contributing to the versatility and adaptability of the docking system.

Figure 4b shows the gender-less docking feature in the design for a better connection between the blocks. The docking mechanism usually has a male on one robot and a female on the other robot, Which allows the robots to lock in only predefined orientations, which will limit the optimization of the docking process. Smorphi’s footprint is 170 mm $\times$ 170 mm square, which allows one robot to dock with another robot on all the sides and all the $90^{\circ }$ orientations.

## Kinematic modeling

The relation between the wheels and the center of the robot was derived from the analysis of the motion of the mecanum wheels. The direction of the wheel rotation varies with the direction and motion of the robot, which enables the holonomic motion of the robot, unlike the standard wheels. The relation between the robot and the wheels is calculated so the velocity controls of the robot can be used to control the robot using the reverse kinematics. Here, *M* is the robot’s width from center to top left, *q* represents the distance of the wheel from the center, and *R* symbolizes the wheel radius. The relationship between the robot’s liner velocity in x direction $V_{x}$ and the angular velocities of each wheel $\omega _{RF}$ (right front wheel), $\omega _{RB}$ (right back wheel), $\omega _{LF}$ (right back wheel), and $\omega _{LB}$ (left back wheel) is given in (1). Similarly, the relationship between the robot’s liner velocity in y direction $V_{y}$ and the angular velocities of each wheel is given in (2).

1 $$\begin{aligned} & V_{x} = \frac{R}{4}(\omega _{RF}+\omega _{RB}+\omega _{LF}+\omega _{LB}) \end{aligned}$$

2 $$\begin{aligned} & V_{y} = \frac{R}{4}(\omega _{RF}-\omega _{RB}-\omega _{LF}+\omega _{LB}) \end{aligned}$$

**Fig. 5:**
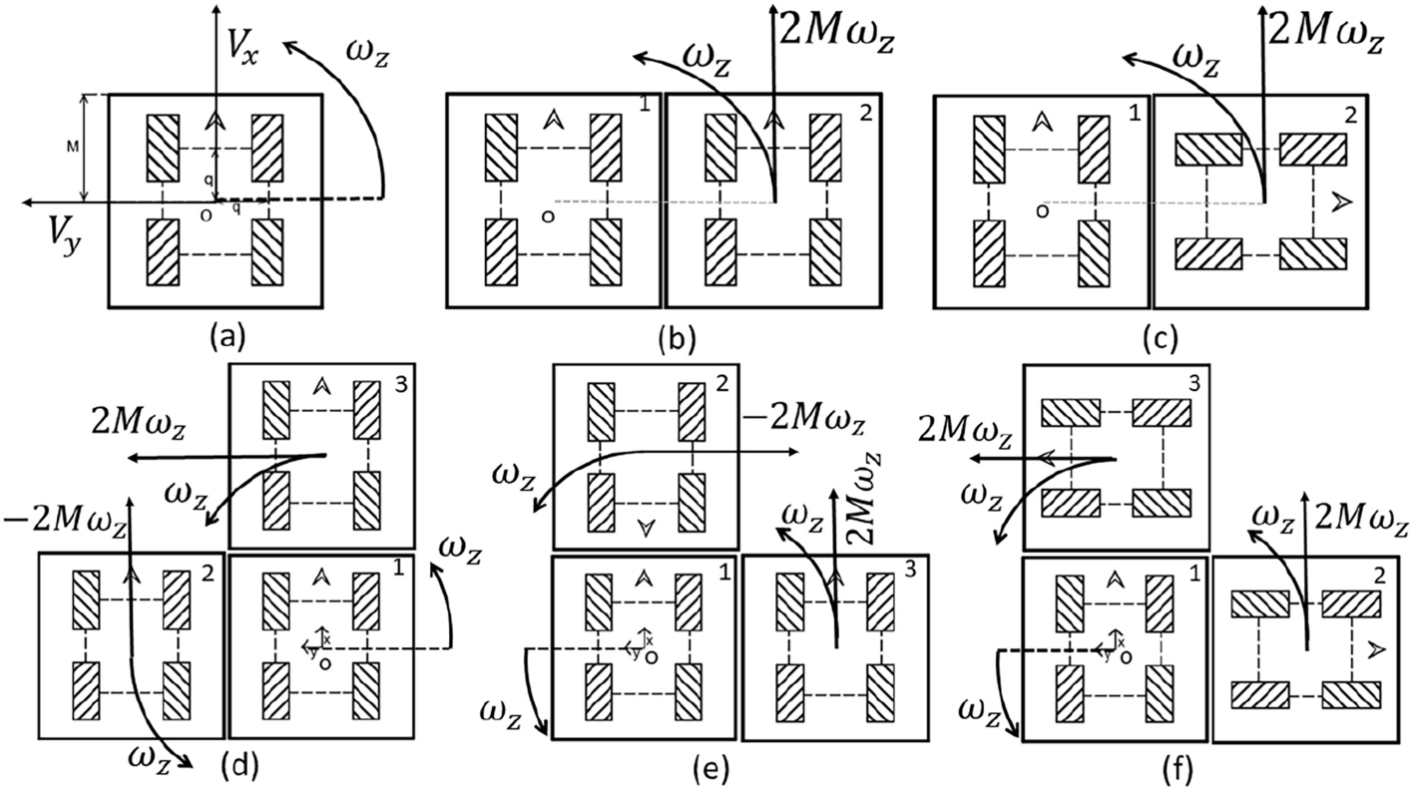
Different combinations of wheel arrangements for kinematics modeling.

Robot rotating around center of robot “o” with angular velocity $\omega _{z}$ is represented in 3. Forward kinematics of the robot rotating around the center of robot “o” (see Fig. 5a) with angular velocity $\omega _{z}$ is given in (4). Here, H is the transformation matrix from robot velocities to wheel angular velocities, and $H^+$ is the inverse transformation of wheel angular velocity to robot motion (refer to 5).

3 $$\begin{aligned} & \omega _{z} = \frac{R}{4}\left( \frac{\omega _{RF}}{2q}-\frac{\omega _{LF}}{2q}-\frac{\omega _{LB}}{2q}+\frac{\omega _{RB}}{2q}\right) \end{aligned}$$

4 $$\begin{aligned} & \begin{bmatrix} V_{X}\\ V_{Y}\\ \omega _{z} \end{bmatrix} = \frac{R}{4} \begin{bmatrix} 1 & 1 & 1 & 1\\ 1 & -1 & 1 & -1\\ \frac{1}{2q} & -\frac{1}{2q} & -\frac{1}{2q} & \frac{1}{2q} \end{bmatrix} \begin{bmatrix} \omega _{RF}\\ \omega _{LF}\\ \omega _{LB}\\ \omega _{RB} \end{bmatrix} \end{aligned}$$

5 $$\begin{aligned} & \begin{bmatrix} V_{x}\\ V_{y}\\ \omega _{z} \end{bmatrix} = H \begin{bmatrix} \omega _{RF}\\ \omega _{LF}\\ \omega _{LB}\\ \omega _{RB} \end{bmatrix},\ \begin{bmatrix} \omega _{RF}\\ \omega _{LF}\\ \omega _{LB}\\ \omega _{RB} \end{bmatrix} = H^+ \begin{bmatrix} V_{x}\\ V_{y}\\ \omega _{z} \end{bmatrix} \end{aligned}$$

Figure 5b,c depict the two different ways of docking two robots. The reference block of the robot center of robot ‘o’. Similarly, the different wheel arrangements of docking with three robots are shown in Fig. 5d–f. Figure 5c, linear motions 1st robot have the $V_{x1} = V_{x}$ and $V_{y1} = V_{y}$ where for 2nd robot $V_{x2} = -V_{y}$ and $V_{y2} = V_{x}$. When the robot rotates around the origin ‘o’ velocities for $1^{st}$robot $\omega _{z1} = \omega _{z}$ and for $2^{nd}$robot $v_{y2} = 2M\omega _{z}$ and $\omega _{z2} = \omega _{z}$.

Similarly for the Fig. 5e, system rotating around origin ‘o’ the velocities of the $1^{st}$robot $\omega _{z1} = \omega _{z}$, for $2^{nd}$robot $v_{y2} = -2M\omega _{z}$ and $\omega _{zb} = \omega _{z}$ and fro $3^{rd}$robot $v_{xc} = 2M\omega _{z}$ and $\omega _{zc} = \omega _{z}$.

**Fig. 6:**
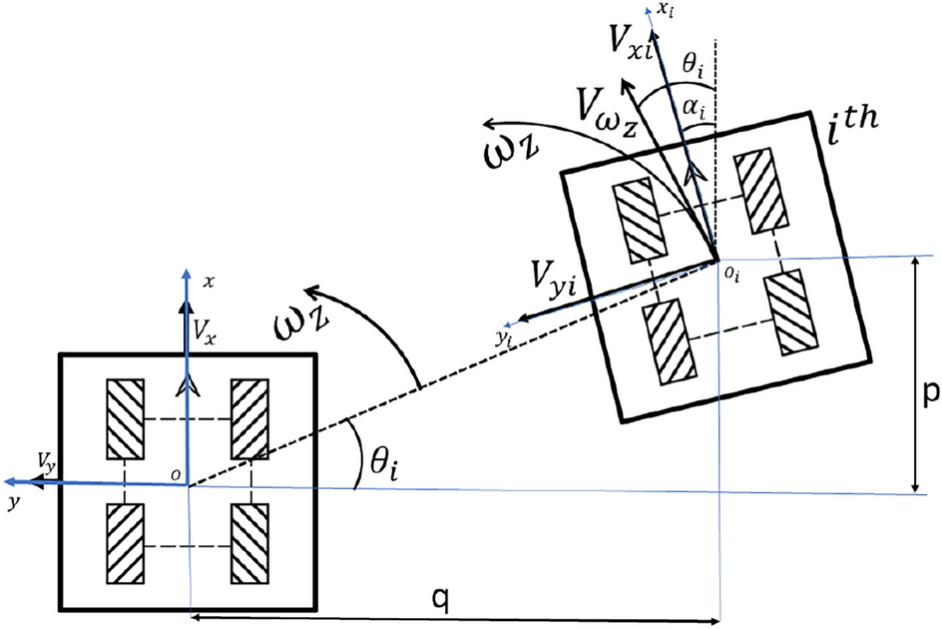
Proposed generalized kinematic model.

Modular robots can be a combination of *n* number of robots during docking. Therefore, we need a generic kinematic model to represent when *n* number of robots are docking. Figure 6 depicts where the kinematics derivation for $i^{th}$ robot docking from the system’s origin. The orientation and distance are known from the IMU and encoder readings respectively. The robot position can be simulated from the data shown in Fig. 6. The known data of the $i^{th}$robot are velocities given to the robot at the origin, orientation, and distance of the robot center position from the origin. The $i^{th}$robot have linear velocity in different orientation of the robot x direction the equivalent $V_{x}$ and $V_{y}$ are $V_{x} = V_{x}\cos {\alpha } + V_{y}\sin {\alpha }$ and $V_{y} = V_{y}\cos {\alpha } - V_{x}\sin {\alpha }$.

In Fig. 6$V_{\omega z} = \omega _{z}\sqrt{i^2 + j^2}$ and including this $V_{xi} = \omega _{z}\sqrt{i^2 + j^2}\cos {(\theta - \alpha )}$ and $V_{yi} = \omega _{z}\sqrt{i^2 + j^2}\sin {(\theta - \alpha )}$ factors to the equivalent $V_{xi}, V_{yi} and \omega _{zi}$ the the transformation matrix (6) is derived.

6 $$\begin{aligned} \begin{bmatrix} V_{xi}\\ V_{yi}\\ \omega _{zi} \end{bmatrix} = \begin{bmatrix} \cos {\alpha } & \sin {\alpha } & \sqrt{i^2 + j^2}\cos {(\theta - \alpha )}\\ -\sin {\alpha } & \cos {\alpha } & \sqrt{i^2 + j^2}\sin {(\theta - \alpha )}\\ 0 & 0 & 1 \end{bmatrix} \begin{bmatrix} V_{x}\\ V_{y}\\ \omega _{z} \end{bmatrix} \end{aligned}$$

## Optimum location for modular robots to assembly

Modular robots play a crucial role in various applications, including logistics, exploration, and firefighting, necessitating assembly or disassembly for specific tasks. Prior to assembly, these modular robots are scattered across different locations within a given environment, which may feature numerous obstacles. Achieving optimal assembly and docking zones becomes essential under these conditions. Considering the diverse locations of the modular robots and the presence of obstacles, it is imperative to determine the optimum zone for assembly while considering factors such as minimizing the total distance traveled and individual distances during the assembly process. The overall energy usage of the robotic system depends on the total distance traveled by the robots. Therefore, the total travel distance should be minimized to reduce the overall energy usage^26^. The robots in a modular robotic system are powered individually by their batteries. To maintain all the robots in approximately the same battery level, the energy usage of individual robots should be the same. In this regard, the individual travel distances should be approximately equal to make the battery drain the same. Thus, the individual distances should be equalized and minimized apart from the total distance. Furthermore, the optimum zone decided to assemble should have sufficient space to gather all the robots and assemble. These considerations should be accounted for when determining the optimum zone for assembly. Addressing such optimization challenges often involves the application of metaheuristic methods.

Among these methods, Genetic Algorithm (GA) emerges as a popular choice^27^. Inspired by the principles of human evolution, GA proves effective in solving complex optimization problems associated with modular robot assembly. By mimicking the process of natural selection, crossover, and mutation, GA aids in identifying optimal path configurations, contributing to the efficient and effective assembly of modular robots in diverse and obstacle environments. Since the optimization problem that we are solving consists of two objectives multi-objective GA^28^ has been used. The cost for the total distance to be navigated by the modular robots, $Cost_d$, is calculated as in (7), where $d_i$ is the A* distance between the assembly location and $i^{th}$ modular robot location. *n* is the total number of modular robots. A* algorithm^29^ is used to calculate the distances without collisions in complex obstacle-surrounding environments. If no path exists, $Cost_d$ tends toward a high value, represented by assigning a very large value. Therefore, the total distance cost of all the modular robot paths is defined in (7), where $P_1$ is a very large positive constant given as penalty when no paths exist for a robot. The second objective, $Cost_{\delta }$, is calculated in such a way that each robot tries to maintain an equal travel distance as given in (8). The A* distance difference with the $i^{th}$ and ${i-1}^{th}$ is denoted as $(d_{i-1}-d_i)$. Here also, if no path exists, a large positive value, $P_2$ is given.

7 $$\begin{aligned} & Cost_d = {\left\{ \begin{array}{ll} \sum \nolimits _{i = 1}^n d_i, & \text { if path exists } \forall i \\ P_{1}, & ~\text {otherwise} \end{array}\right. } \end{aligned}$$

8 $$\begin{aligned} & Cost_\delta = {\left\{ \begin{array}{ll} \sum \nolimits _{i = 2}^n (d_{i-1}-d_i)^2, & \text { if path exists } \forall i \\ P_{2}, & ~\text {otherwise} \end{array}\right. } \end{aligned}$$

**Fig. 7:**
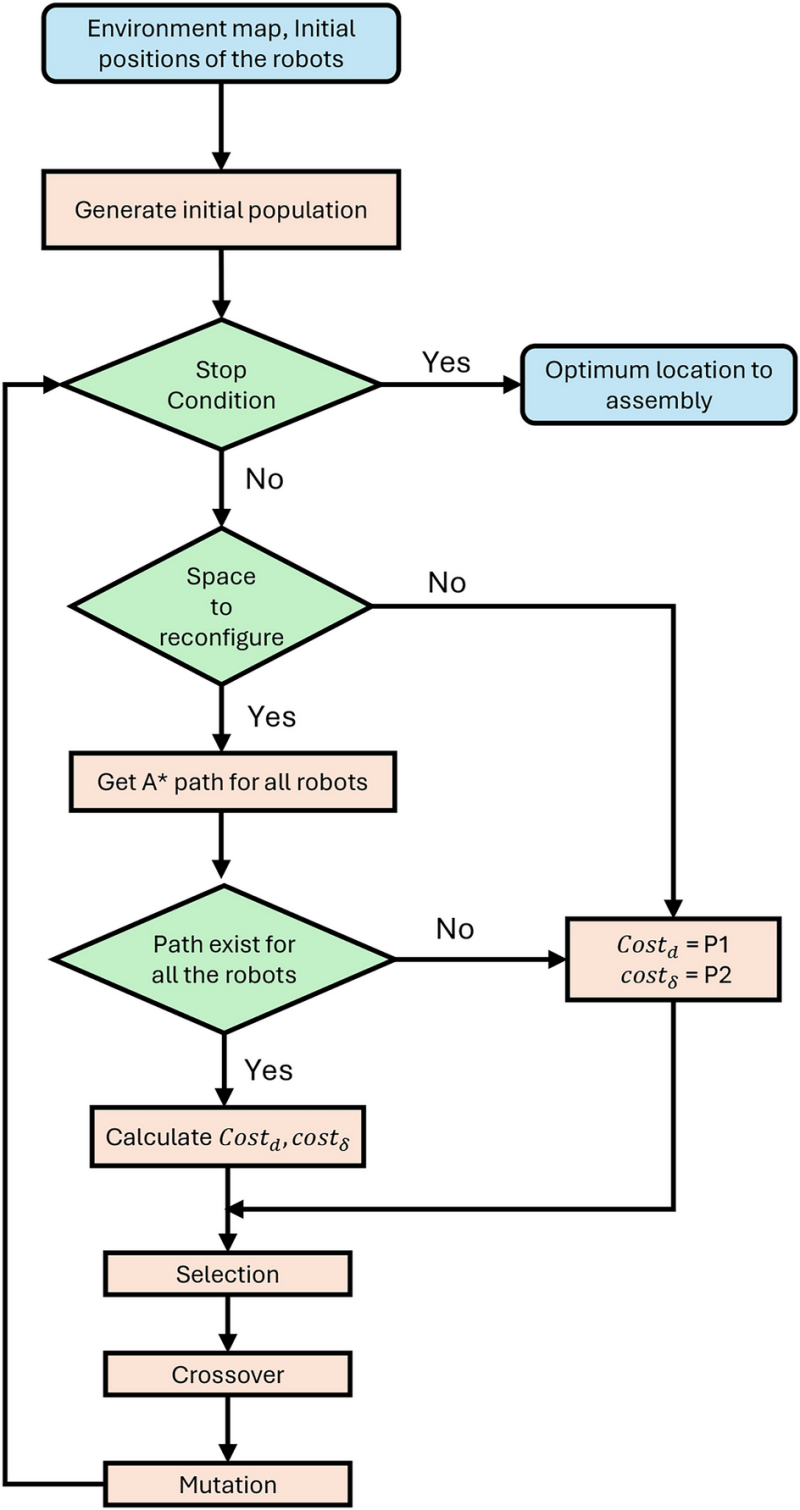
Determining optimum location for modular robots to assembly modeling.

The proposed method is outlined in Fig. 7. The environment map is created by utilizing the Lidar information. The initial positions of the robots are taken as inputs. The initial positions of the robots are provided as inputs, which can be obtained from each modular robot’s Lidar and localization data. The multi-objective genetic algorithm generates an initial population of random solutions for the optimum location within the search space. If the A* distance from each robot to the generated assembly location exists and the space is sufficient for docking, $Cost_d$ and $Cost_{\delta }$ are calculated. Otherwise, penalty $P_1$ and $P_2$ are assigned for the cost functions, respectively. Following this step, the best-fit individuals within the current solution are chosen. Subsequently, crossover and mutation are used to produce the succeeding generation of the population. This iterative process persists until a predetermined stopping criterion is satisfied. Given the nature of multi-objective optimization, a collection of Pareto optimal solutions emerges upon termination. From this set of solutions, the knee point solution, representing the optimal compromise where viable paths are available, is chosen as the ultimate solution for the assembly point.

## Experimental results

### Kinematic model validation

**Fig. 8:**
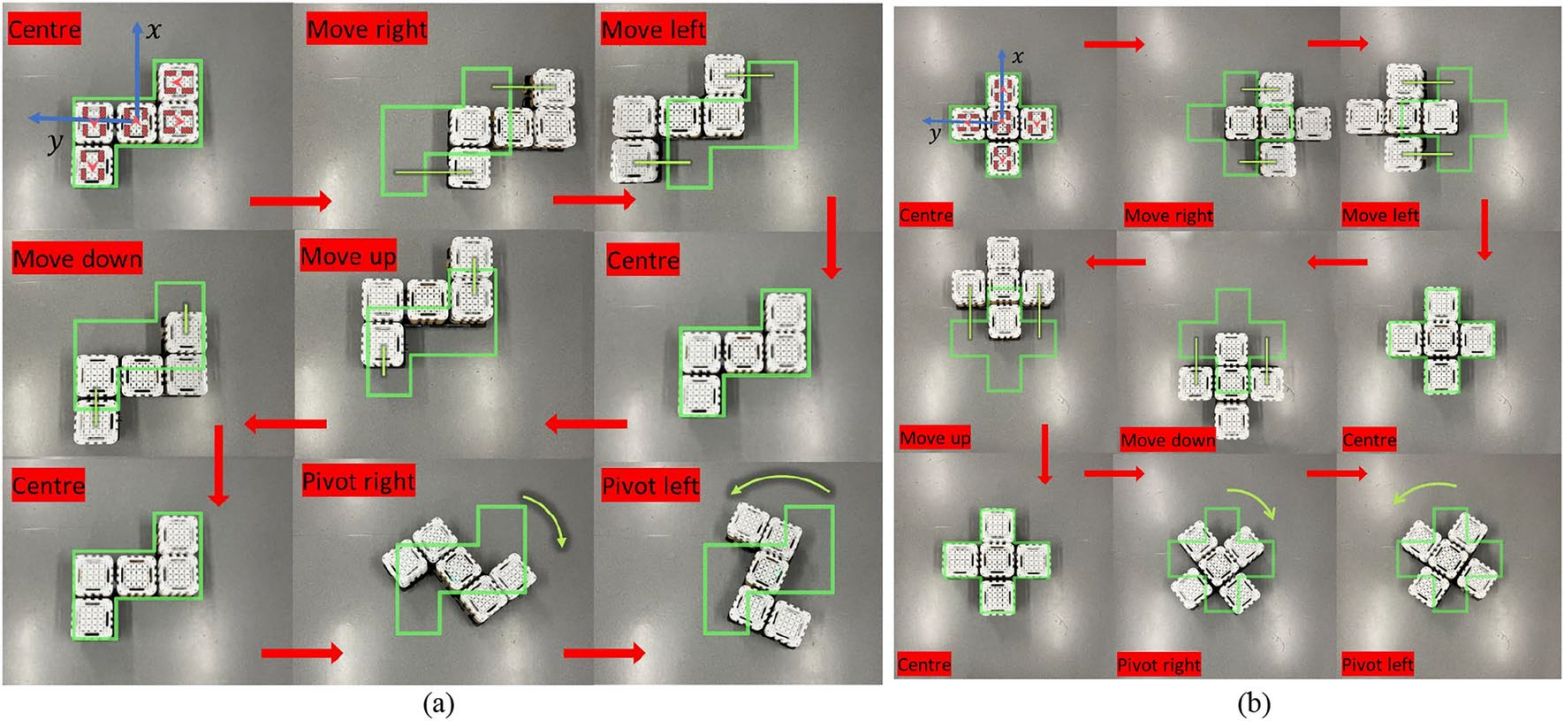
Kinematic model testing with five robots with different shapes. The wheel arrangements of each modular robot are shown in the first figure of the sequence. (**a**) Kinematic model testing for shape 1. (**a**) Kinematic model testing for shape 2.

The proposed generic kinematic model for mecanum wheel modular robots has been tested with real-time experiments. Two different configurations with five modular robots have been used. Figure 8 depicts two test cases used in the study. The first image of the sequence in both cases illustrates the wheel configuration of each robot alongside the reference block. The green frame highlights this as the reference position for both scenarios. Movement and rotations of the robot are depicted with reference to this green frame. In both instances, the robot exhibits smooth motion in forward, backward, left, and right directions and clockwise and counterclockwise rotations. These two experimental cases demonstrate the practical application and effectiveness of the derived kinematic model in enabling the robots to configure themselves into different shapes as required by the system. The Smorphi robotic system’s adaptability and versatility are demonstrated by the robots’ capacity to generate the kinematics for every robot unit by precisely getting orientation and location feedback.

### Determining optimum zone for assembly

#### Simulation results

The proposed method has been simulated under diverse scenarios to evaluate the performance. The multi-objective Genetic Algorithm (GA) parameters were adjusted in such a way as to have a better performance. The population size was 50 since the number of variables is two. The intermediate crossover method with a fraction of 0.8 has been used. Pareto fraction, selection method, distance measure, and mutation function were 0.35, tournament, phenotype crowding, and adaptive feasible, respectively. Function evaluation of 2500 is taken as the stopping criteria.

**Fig. 9:**
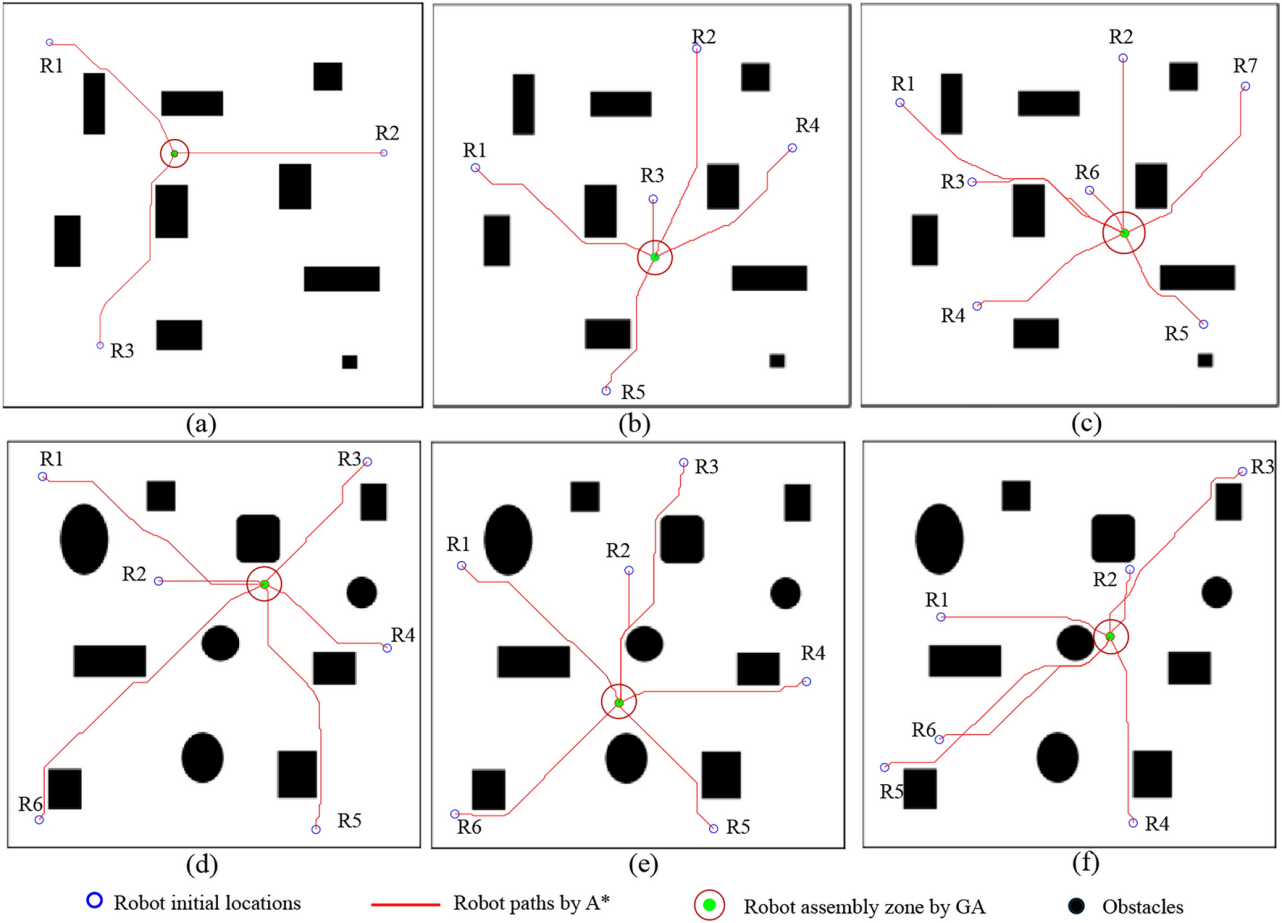
The optimum paths and optimum goal location for assembly for modular robots.

**Fig. 10:**
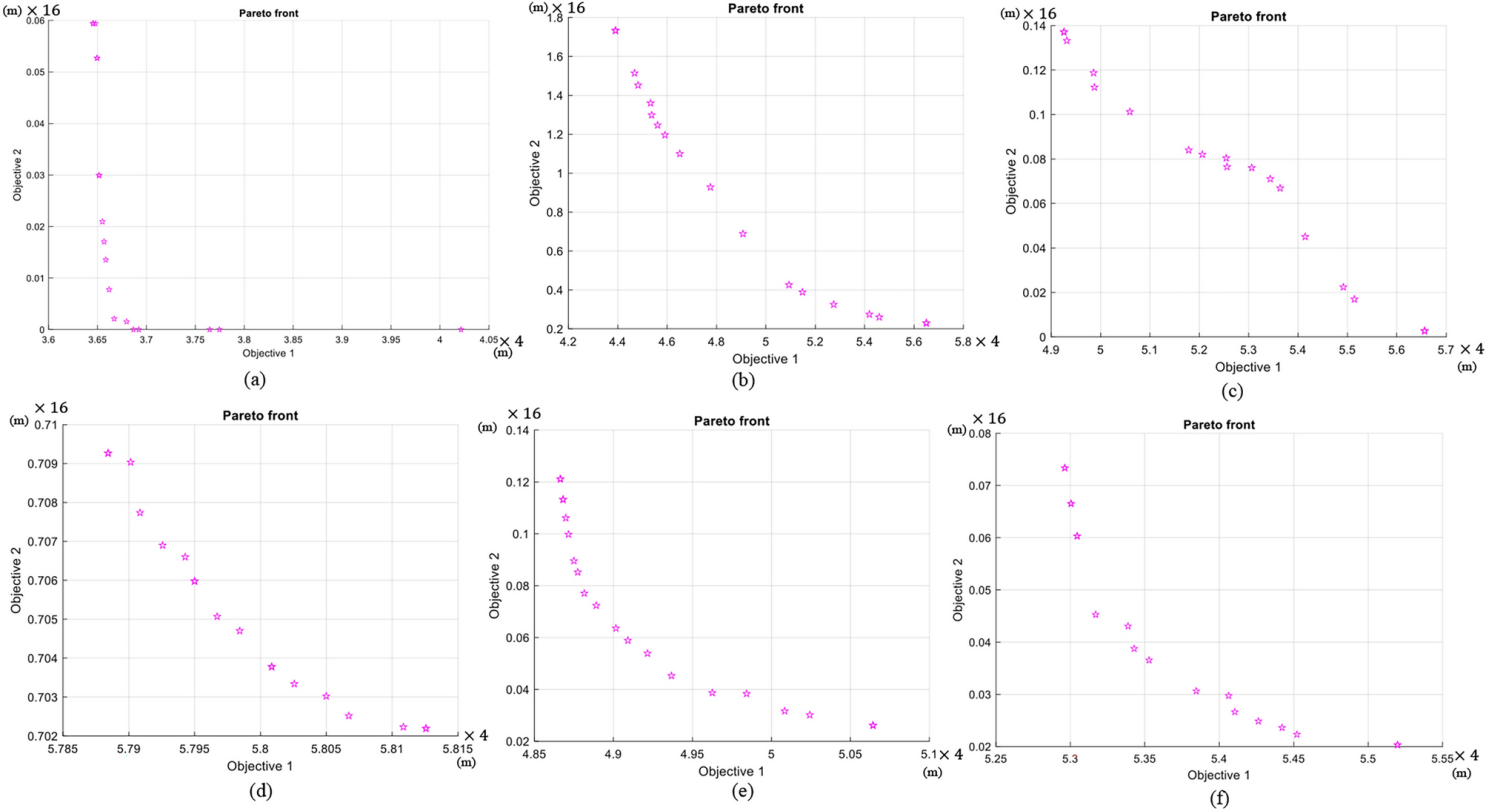
Pareto front retruned from GA for all the cases considered in the simulation. Objective 1: $Cost_D$ and Objective 2: $Cost_\delta$.

The environment size of 10 m $\times$ 10 m consisting of heterogeneous obstacles was considered for simulations. Figure 9a–c show the increase of robots such as 3, 5, and 7 for the same environments, and the optimum assembly point is shown here. Here, the assembly location is arranged in such a way that it considers a zone around the optimum assembly point to have space for assembling, considering the number of robots. Figure 9d–f show 6 robots determining an optimum location for assembling with each case of changing the initial positions of all the robots. The Pareto front for all six cases is shown in Fig. 10. Two objectives, the total distance and the difference of the distances, are indicated in the axis in meters, and the axis values should be multiplied by four times since the cases were simulated in resizing the environment of a 4:1 ratio. The cost values of $Cost_d$ and $Cost_\delta$ for each case are included in Table 1. The method based on GA has been compared with a multi-objective Pattern Search (PS)^30^ and a random assembly point generator, considering the assembly point of obstacle-free and reconfiguring space requirement. In case (a), using the GA, $Cost_d$ has received as 14.7 m and $Cost_\delta$ obtained as 0.0084 m$^2$. For the same case using PS, the $Cost_d$ and $Cost_\delta$ are received as 14.7 m and 0.0880 m$^2$, while the $Cost_d$ and $Cost_\delta$ of the random generator are observed as 23.6 m and 0.0464 m$^2$. These cost values are comparatively larger than the GA results. Similarly, the proposed GA method costs are less than the PS and random method for all the simulation cases.

**Table 1:** Cost values obtained from GA, PS, and random assembly locations.

Case	GA		PS		Random	
	$Cost_d$ (m)	$Cost_\delta$ (m$^2$)	$Cost_d$ (m)	$Cost_\delta$ (m$^2$)	$Cost_d$ (m)	$Cost_\delta$ (m$^2$)
a	14.7	0.0336	14.7	0.0880	23.6	0.0464
b	19.6	11.0160	21.0	5.2352	33.9	54.2112
c	21.7	0.7216	25.4	3.4384	34.7	64.2464
d	23.2	11.2752	31.0	14.8640	33.8	38.5600
e	19.7	0.8624	28.4	2.7904	46.3	124.4512
f	21.5	0.4896	27.2	5.7744	35.9	199.1344

#### Modular robot hardware experiments

The experiments were performed using the robot hardware to validate the real world applicability. Only the proposed method based on GA has been considered here since it has shown the superior performance than others in simulations. The map generated through Lidar scans were used to determine the optimum zone for assembly. In this work, the robot operates with a known map. Based on this pre-existing map, the assembly location is identified through an initial global planning process. The positions of the robots within the environment were accurately determined via localization, which involved sensor fusion from Lidar, IMU, and wheel odometry data. Two distinct environments were employed for the experiments: one accommodating three robots and the other accommodating five robots.

**Fig. 11:**
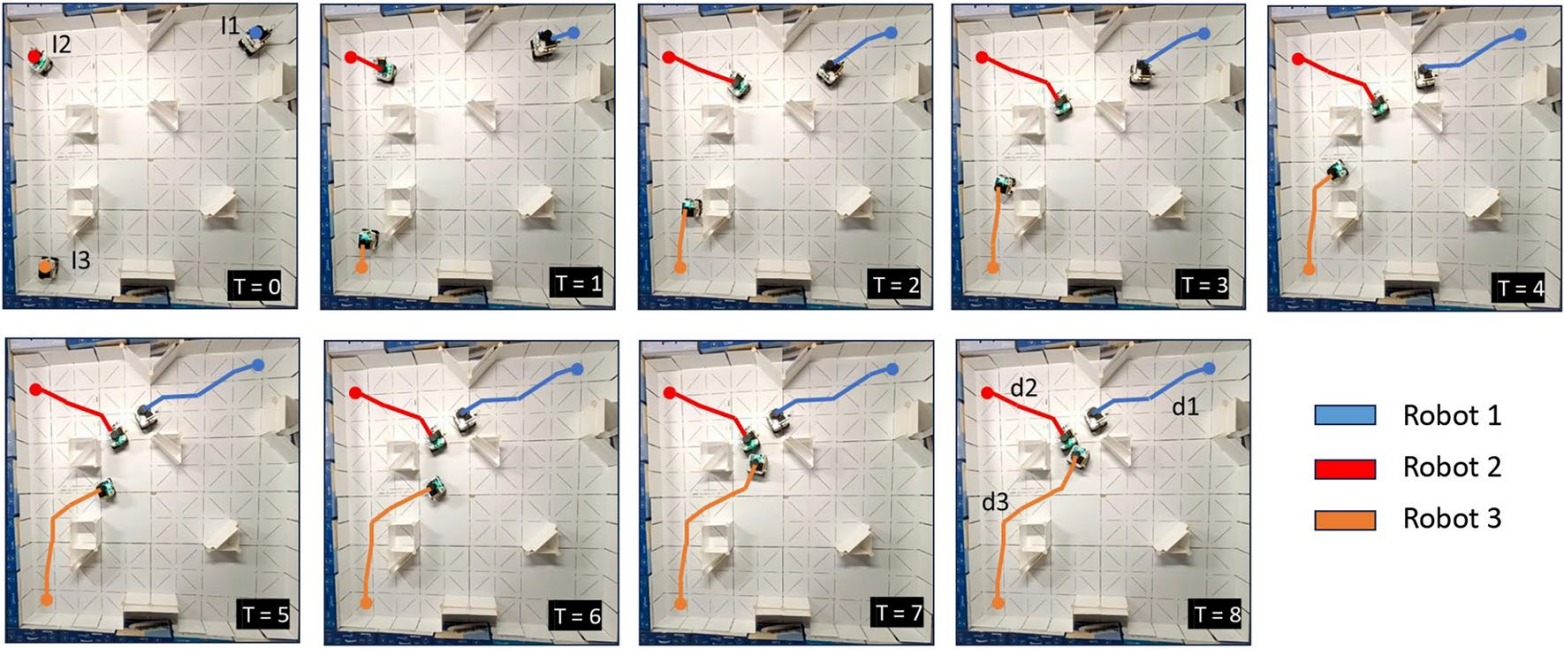
Real-time hardware results with 3 robots in a 2.50 m $\times$ 2.50 m environment.

In the scenario with three robots, the environment spanned an area of 2.5 m $\times$ 2.5 m, as depicted in Fig. 11. This environment facilitated the optimization process tailored to the specific objectives and constraints of the experiment. Robots 1, 2, and 3 started from initial points $I_1 (0.32, 0.49)$, $I_2 (0.48, 2.26)$, and $I_3 (2.27, 2.33)$, respectively. The paths generated from the proposed method for each robot are annotated in the figure. The distances of robots 1, 2, and 3 were received as $d_1 = 1.21$ m, $d_2 = 0.89$ m, and $d_3 = 1.85$ m, respectively. The total travel distance $Cost_d$ and disparity distance $Cost_\delta$ for this case were 3.95 m and 1.0064 m$^2$, respectively.

**Fig. 12:**
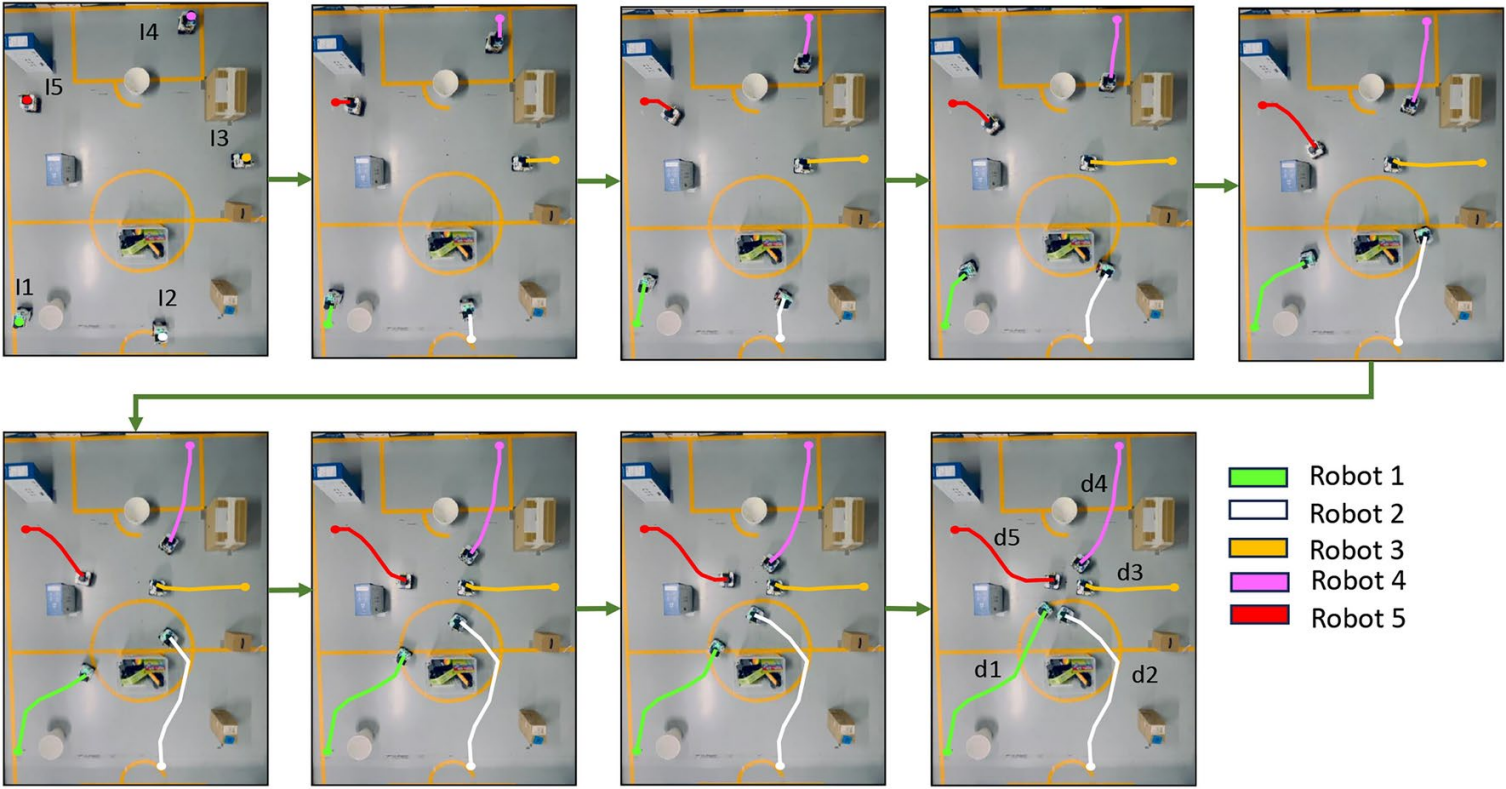
Real-time hardware results with 5 robots in a 3.40 m $\times$ 4.30 m environment.

Similarly, in the scenario with five robots, as in Fig. 12, details regarding the environment’s dimensions were 4.30 m $\times$ 3.40 m. This environment likely provided a larger space to accommodate the increased number of robots, allowing for more complex interactions and challenges. Robots 1, 2, 3, 4, and 5 started from initial points $I_1 (0.39, 0.37)$, $I_2 (0.52, 0.206)$, $I_3 (2.49, 3.10)$, $I_4 (3.93, 2.22)$, and $I_5 (3.11, 0.52)$ respectively and followed the paths shown in Fig. 12. The distance of each robot calculated are $d_1$ = 2.31 m, $d_2$ = 2.32 m, $d_3$ = 1.12 m, $d_4$ = 1.55 m, and $d_5$ = 1.54 m for robots 1, 2, 3, 4 and 5 respectively. The total travel distance $Cost_d$ and disparity distance $Cost_\delta$ for this case were 8.84 m and 1.6219 m$^2$, respectively.

By conducting experiments in these varied environments, the performance and effectiveness of the robotic systems were thoroughly evaluated under different conditions. These experimental results validated the applicability of the proposed method for determine the optimum assembly zone in real world scenarios.

## Conclusion

Reconfigurable modular robots are used in logistics, firefighting, and exploration applications. The robots are required to navigate to an assembly location and do docking to perform tasks that requires higher strength. This paper proposed a novel method for determining an optimum assembly zone for different numbers of reconfigurable modular robots using multi-objective GA in different obstacle environments. This optimization minimizes the total travel distance and the difference between the individual distances. The path determined by the A* algorithm is considered here for distances. Furthermore, the paper introduced a generic kinematic model enabling holonomic navigation with any assembled shape.

Hardware experiments have been conducted considering a set of different reconfigurations to validate the proposed kinematic model. The proposed kinematic model was able to perform holonomic navigations. Simulations and actual robot experiments have been conducted to evaluate the performance of determining the assembly zone for modular robots. According to the experimental results, the proposed method based on GA can determine an assembly zone with lesser total distance and similar individual distances compared to PS and random selection. The proposed algorithm currently only considers static obstacles during navigation. The determining of an optimum assembly zone, considering the dynamic obstacles in the environment, is considered for future work. The docking mechanism proposed in this paper is manual, and autonomous docking is proposed as future work.

## Data Availability

The datasets generated during and/or analysed during the current study are available from the corresponding author on reasonable request.
